# UAV hyperspectral analysis of secondary salinization in arid oasis cotton fields: effects of FOD feature selection and SOA-RF

**DOI:** 10.3389/fpls.2024.1358965

**Published:** 2024-02-19

**Authors:** Zeyuan Wang, Jianli Ding, Jiao Tan, Junhao Liu, Tingting Zhang, Weijian Cai, Shanshan Meng

**Affiliations:** ^1^ College of Geography and Remote Sensing Sciences, Xinjiang University, Urumqi, China; ^2^ Xinjiang Key Laboratory of Oasis Ecology, Xinjiang University, Urumqi, China; ^3^ Key Laboratory of Smart City and Environment Modelling of Higher Education Institute, Xinjiang University, Urumqi, China

**Keywords:** precision agriculture, UAV hyperspectral, fractional-order differentiation, feature variables, SOA-RF

## Abstract

Secondary salinization is a crucial constraint on agricultural progress in arid regions. The specific mulching irrigation technique not only exacerbates secondary salinization but also complicates field-scale soil salinity monitoring. UAV hyperspectral remote sensing offers a monitoring method that is high-precision, high-efficiency, and short-cycle. In this study, UAV hyperspectral images were used to derive one-dimensional, textural, and three-dimensional feature variables using Competitive adaptive reweighted sampling (CARS), Gray-Level Co-occurrence Matrix (GLCM), Boruta Feature Selection (Boruta), and Brightness-Color-Index (BCI) with Fractional-order differentiation (FOD) processing. Additionally, three modeling strategies were developed (Strategy 1 involves constructing the model solely with the 20 single-band variable inputs screened by the CARS algorithm. In Strategy 2, 25 texture features augment Strategy 1, resulting in 45 feature variables for model construction. Strategy 3, building upon Strategy 2, incorporates six triple-band indices, totaling 51 variables used in the model’s construction) and integrated with the Seagull Optimization Algorithm for Random Forest (SOA-RF) models to predict soil electrical conductivity (EC) and delineate spatial distribution. The results demonstrated that fractional order differentiation highlights spectral features in noisy spectra, and different orders of differentiation reveal different hidden information. The correlation between soil EC and spectra varies with the order. 1.9th order differentiation is proved to be the best order for constructing one-dimensional indices; although the addition of texture features slightly improves the accuracy of the model, the integration of the three-waveband indices significantly improves the accuracy of the estimation, with an R^2^ of 0.9476. In contrast to the conventional RF model, the SOA-RF algorithm optimizes its parameters thereby significantly improving the accuracy and model stability. The optimal soil salinity prediction model proposed in this study can accurately, non-invasively and rapidly identify excessive salt accumulation in drip irrigation under membrane. It is of great significance to improve the growing conditions of cotton, increase the cotton yield, and promote the sustainable development of Xinjiang’s agricultural economy, and also provides a reference for the prevention and control of regional soil salinization.

## 1 Introduction

Soil salinization, notably secondary salinization on mulched irrigated land, is a mounting global issue significantly constraining agricultural development (Wang et al., 2023). Currently, secondary salinization in Xinjiang is highly severe and shows an inclination for further escalation (Hua et al., 2019). The alarming ecological degradation poses a significant threat to oasis agricultural security, profoundly impacting regional socio-economic development (Yu et al., 2022). Cotton is the pillar industry of Xinjiang’s eight industrial clusters, accounting for more than 90% of the national cotton industry, is China’s important strategic reserves again resources (Liwen et al., 2019). About 1.6 million hectares of cotton fields in Xinjiang are irrigated with drip irrigation, and the area is still increasing (Hou et al., 2022), large-scale ground film dripping in the short-term yield increase benefits produced after the secondary salinization is serious, and the soil secondary salinization is increasing (Wang et al., 2011). In the oasis ecosystem with farmland as the main body, under the technical means of membrane-covered drip irrigation, it leads to the vegetation canopy again weakening the pure soil information that is obscured, and it is difficult to dig out the important information related to the soil, especially in the interference or influence of water-saving conditions of drip irrigation under the membrane, and it is difficult to intelligently and rapidly extract the secondary salinization as the weak information. Therefore, it is imperative to design a novel monitoring method for secondary salinization of soil. This method should be able to obtain highly accurate, extensive and rapid information on secondary soil salinization to provide scientific support for the management of secondary soil salinization and the development of precision agriculture.

Obtaining accurate information on soil salinity in cotton fields is challenging due to the presence of mulch, which makes it impossible to directly acquire electromagnetic spectrum images of the soil surface through remote sensing (Ding et al., 2013). Soil salinity accumulation hinders effective water uptake by the cotton root system (Zhang et al., 2017). Simultaneously, the near-infrared and mid-infrared bands exhibit high sensitivity to the moisture content of cotton leaves (Arshad et al., 2018). Additionally, spectral reflectance of cotton leaves progressively rises with increasing soil salinity (Zhang et al., 2012). For indirect soil salinity monitoring, selecting spectral bands closely linked to cotton plant growth as characteristic bands allows for obtaining richer spectral features through band combinations. This approach facilitates the use of spectral data to infer soil salinity.

Prior studies commonly utilized conventional satellite-mounted multispectral sensors or synthetic aperture radar (SAR) to acquire remotely sensed imagery to invert soil salinity (Wang J. et al., 2019). Despite their easy accessibility, these data sources often exhibit drawbacks like low spatial (Tan et al., 2023) and spectral resolution alongside lengthy revisit periods (Fan et al., 2021). While the lower spatial resolution and longer revisit period are advantageous for soil salinity detection across vast regions (Sahbeni et al., 2023), precision agriculture requires a method apt for monitoring soil salinity in smaller areas. Conversely, traditional multispectral imagery, due to fewer bands, covers a limited spectral range. The broader bands frequently lack specificity and fail to provide sufficient spectral information to delineate abnormalities caused by salt stress on crops (Huang et al., 2021). The hyperspectral data, comprising multiple consecutive bands, facilitate the spectral characterization of distinct bands (Borsoi et al., 2021), aiding in establishing the correlation between soil salinity and crop canopy spectral response. This facilitates extracting anomalous information and enables monitoring soil salinity in cotton fields by linking this anomalous information to soil salinity. Recently, due to the proliferation of UAV technology in commercial applications and enhanced UAV capabilities, low-altitude imaging platforms equipped with hyperspectral sensors on UAVs have found extensive utilization across various research domains (Zhang et al., 2021), encompassing precision agriculture (Daponte et al., 2019), biomass estimation (Jones et al., 2020), water quality monitoring (Sibanda et al., 2021), and forest protection (Jiménez López and Mulero-Pázmány, 2019), among other fields. Imagery obtained through UAV platforms equipped with hyperspectral sensors achieves accuracy at the centimeter level, proving to be an efficient, cost-effective, and real-time method for monitoring salinity in small-scale agricultural fields (Paul et al., 2022).

Hyperspectral data encompass numerous spectral bands, often displaying high redundancy, necessitating band selection to diminish data dimensionality and enhance information extraction efficiency (Sun and Du, 2019). Competitive Adaptive Reweighted Sampling (CARS) is a widely used method in hyperspectral band selection (Lei et al., 2022). This method combines competitive learning and adaptive sampling strategies aimed at selecting the most informative spectral bands for downscaling and feature selection of hyperspectral data. The CARS method has achieved excellent performance on many hyperspectral datasets, and the accuracy and efficiency of feature selection can be significantly improved by selecting the most relevant bands. The Band Combination Index (BCI) method is a powerful tool for constructing optimal band feature combinations (Liu et al., 2023), which helps to improve the efficiency of information extraction and analysis of hyperspectral data by selecting the optimal band combinations. Although these spectral processing methods have been widely used, there are fewer cases of applying them to hyperspectral inversion of soil salinity, and the prediction is still unclear. Texture features as model variables can add information about spatial distribution to the model, and some studies have demonstrated that texture information in UAV-based imagery has great potential for crop parameter monitoring (Li et al., 2019), but there have been few studies on the use of texture features for the estimation of soil salinity.

Prediction of soil properties by linking spectral information to soil properties through mathematical modeling is already a well-established scheme (Beattie and Esmonde-White, 2021). The Random Forest (RF) model is an integrated learning algorithm that makes predictions by constructing multiple decision trees. Each decision tree is constructed based on a training dataset obtained from random sampling and features are randomly selected for partitioning at each node (Sheykhmousa et al., 2020). The final prediction results are synthesized from the predictions of all the decision trees and have been widely used for regression prediction of soil attributes by virtue of its excellent ability to handle nonlinear relationships. However, the RF algorithm itself suffers from some problems, such as susceptibility to overfitting and sensitivity to parameters (Beattie and Esmonde-White, 2021). To address these problems, the Seagull Optimization Algorithm (SOA) was introduced to optimize the RF algorithm. SOA is a bionic intelligence algorithm that simulates the foraging behavior of seagulls. It optimizes the performance of the algorithm through the search and learning process in the foraging behavior (Dhiman et al., 2021). SOA is used to optimize parameter selection and feature selection for RF algorithms.

The main research objectives of this study are (1) to investigate the effect of FOD treatment on drone hyperspectra and to explore how the correlation between plant canopy spectra and soil salinity varied with order after treatment with FOD; (2) to construct and screen the single-band features, texture features, and triple-band features by using the CARS algorithm, the Boruta algorithm, the grayscale covariance matrix and the BCI algorithm, respectively, and to explore how the sensitivity of these feature variables to the model; (3) to analyze and compare the capabilities exhibited by SOA-RF and traditional RF models in soil salinity prediction.

## 2 Study area and data

### 2.1 Study area and soil samples

**Figure 1:**
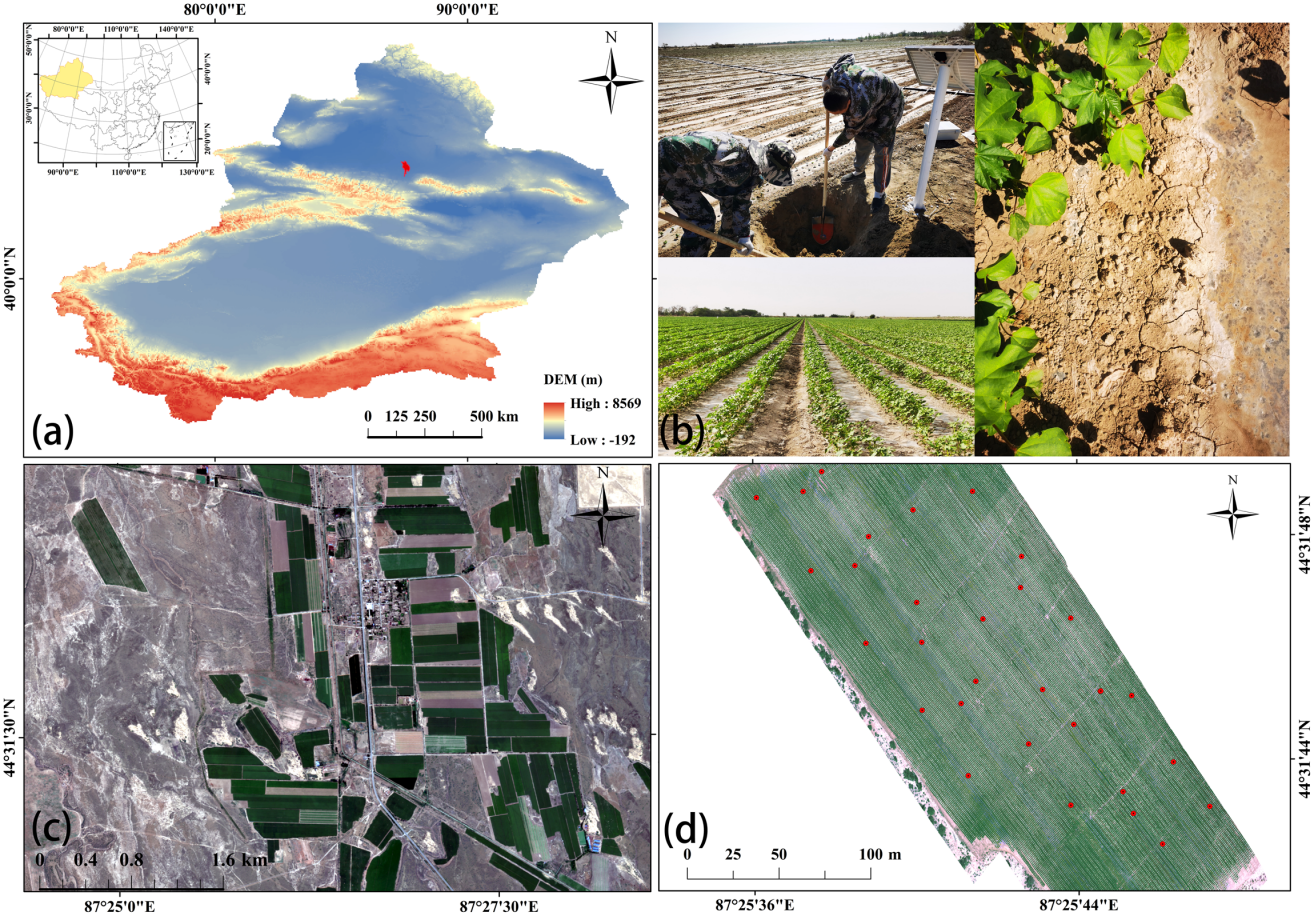
Schematic map of the study area [**(A)** Zoning location of Wujiaqu city; **(B)** Landscape photographs of the study area; **(C)** Schematic map of the location of typical cotton fields; **(D)** Distribution of sampling points].

Wujiaqu City is located in the urban agglomeration of the northern slope of Tianshan Mountain, the northern foothills of Tianshan Mountain and the southern edge of the Junggar Basin (43°59′25″ to 44°39′00″N and 87°17′42″ to 87°43′15″E). As warm and humid air masses are difficult to enter the Xinjiang Basin due to the obstruction of mountain ranges, resulting in scarce precipitation and large temperature variations throughout the year (-38~42°C); scarce precipitation (150mm) and strong evaporation (2000mm). In order to better explore the ability and ubiquity of UAV hyperspectral data to indirectly monitor soil salinization through vegetation spectral information in oasis farmland in arid zones, a field study was conducted in June 2022 in a typical cotton field in Wujiaqu City (**Figure 1**).

The field sampling time was selected on June 12, 2022, which was in the cotton bud stage, with no interference from precipitation, irrigation, or anthropogenic factors, and the unmanned data hyperspectral data were sampled at the same time as the soil samples to ensure the reliability of the measured data. Thirty-three surface (0-10 cm) soil samples were collected using the five-point sampling method, and the coordinates of each sampling point were recorded using a portable GPS device. Soil samples were air-dried and ground in the laboratory, and sieved through a 2-mm sieve. Soil solutions were prepared according to the method of 1:5 soil leachate, and soil parameters such as solution EC_1:5_ and pH were measured using a multiparameter meter (WTWinoLab^®^ Multi3420 set B, WTW GmbH, Germany) at a constant room temperature of 25°C. Soil EC_1:5_ can be used to indicate soil salinity.

### 2.2 Drone data

In order to acquire the UAV hyperspectral data in the study area, a Nano-Hyperspec (Headwall Photonics Inc., Bolton, MA, USA) ultra-miniature airborne hyperspectral imager (UHI) was mounted on a M600 Pro six-rotor UAV flight platform for spectral data acquisition. for spectral data acquisition. The parameters of the hyperspectral imager are shown in **Table 1**. Hyperspectral images with a spatial resolution of 4.4 centimeters and a spectral range of 400~1000 nm can be acquired at a flight altitude of 100 meters. The hyperspectral image data were acquired on June 12, 2022 at 13:00 BST in a clear, cloudless environment with a wide field of view, and a calibrated white cloth with an area of 2.5m×2.5m and a reflectance of 60% was used for reflectance correction and dark current correction. After data acquisition, the hyperspectral images were processed using Hyperspec III (version 1.3) and SpectralView (version 1.0) software for radiance-to-brightness conversion, atmospheric correction, and geometric correction.

**Table 1:** Nano-Hyperspec hyperspectral imager main parameters.

Parameters	Value
Spectral range	400~1000nm
Number of spectral channels	272
Number of space channels	640
Spectral sampling interval	2.2nm/pixel
spectral resolution	2.2nm
Maximum numerical aperture	F/2.5
lens focal length	8mm (12mm)
field of view	32°(22°)
Maximum frame rate	350fps
power wastage	13W
Weight (with head)	<1.3kg

## 3 Methods

### 3.1 Spectral pre-processing

Savitzky-Golay (SG) smoothing is the most efficient way to process spectral reflectance data of saline soils (Ge et al., 2021). The hyperspectral images acquired in this study had a resolution of 4.4 cm and covered 272 bands, and in order to ensure that this dense spectral information can objectively and accurately represent the spectral features of cotton that are subjected to abnormal changes due to salinity, the hyperspectral images were first subjected to Savitzky-Golay (S-G) smoothing with eight points and a polynomial order of nine. Standard normalized variate (SNV) is often used to remove spectral signal variance and is widely used in spectral analysis (Wang H.-P. et al., 2022). In order to correct the spectral errors between samples due to scattering, the hyperspectral images were corrected individually for each spectrum using the SNV. These preprocessing procedures were implemented using the MATLAB R2021b software was implemented.

### 3.2 Fractional-order differentiation

Hyperspectral data contain information from different wavelengths, which may contain noise (Ge et al., 2022). Fractional order derivative (FOD) can be used to smooth and remove this noise, thus improving the quality of the data (Wang J. et al., 2022), and can also be used to enhance features in hyperspectral data (Jiang et al., 2022). Since the G-L definition is relatively concise and has a better performance in terms of for transform and spectral processing (Hong et al., 2019), In this study, the G-L definition form is chosen to process the hyperspectral data, and the G-L definition expression is shown in Equation (1).


(1) $$\begin{array}{c} \frac{d^{\alpha }f\left(\lambda \right)}{d\lambda^{\alpha }}=f\left(\lambda \right)+\left(-\alpha \right)f\left(\lambda -1\right)+\frac{\left(-\alpha \right)\left(-\alpha +1\right)}{2}+\cdots +\frac{\text{Γ}\left(-\alpha +1\right)}{n!\left(-\alpha +1\right)}f\left(\lambda -n\right) \end{array}$$

Where *α* is the order, Γ is the Gamma function, *λ* is the wavelength, and *n* is the D-value of the constraint limit of the differential equation.

### 3.3 Screening and construction of features

#### 3.3.1 Competitive adaptive reweighted sampling

Competitive adaptive reweighted sampling (CARS) is a feature variable selection method that combines Monte Carlo sampling and regression coefficients of the PLSR model, and the algorithm is based on the principle of “survival of the fittest” in Darwinian evolution (Dai et al., 2015), which mainly consists of four steps: (1) Monte Carlo model sampling, the percentage of the absolute value of regression coefficients as the importance of the variables or the interpretability of the target variables; (2) exponential decay wavelength selection, the first stage for rapid selection to eliminate a large number of variables, and the second stage for accurate analysis; (3) adaptive reweighting sampling, resampling according to the number of variables determined in the previous step, to establish a screening-based variables to build an analytical prediction model based on screening, and calculate its cross-validated root mean square error RMSE_CV_; (4) loop iteration, set the number of iterations, and determine the optimal set of variables based on the smallest RMSE_CV_, which is the desired feature variable. In this study, CARS was implemented using MATLAB R2021b software.

#### 3.3.2 Gray-Level Co-occurrence Matrix

Texture features are spatially dimensional information that characterize spatial distribution (Haralick et al., 1973). Band-based estimation models use only one dimension of information, the amount of change, without considering its distribution. The combination of band indices with image texture parameters can increase the dimensionality of the information. Gray-Level Co-occurrence Matrix (GLCM) is one of the important tools used for image texture analysis. GLCM analysis has a wide range of applications in image processing and analysis, especially in the fields of texture recognition, image classification (Sidek, 2015). It can help you describe the spatial relationship between pixels in an image at the gray level and extract various texture features from it. The computational steps of GLCM are as follows: (1) Select a specific direction and distance: determine the image region and direction of interest. Within this region, for each pixel, observe its relationship with neighboring pixels at a specific distance and direction. (2) Construct the cooccurrence matrix: for the selected direction and distance, count the frequency of occurrence between each gray level pixel and its neighboring pixels in the image. This frequency matrix is known as GLCM, and the texture is characterized by four features, namely, contrast, entropy, angular second-order distance, and correlation, computed from the gray-level co-production matrix (**Table 2**), where *P*(*i*,*j*) denotes the frequency of simultaneous occurrences of pixel pairs of gray levels *i* and *j*(*i*, *j* =0, 1,2 3,…, *N*), that is the frequency of gray level cooccurrence matrices were normalized GLCM and image texture measurements were computed using ENVI software and MATLAB R2021b.

**Table 2:** Texture features and their implications.

Textural features	Formula	Implication of the formula
Contrast(Con) (Szantoi et al., 2013)	$C_{\text{OM}}=\sum_{i}\sum_{j}P\left(i,j\right){\left(i-j\right)}^{2}$	Indicates localized changes in the image
Entroy(Ent) (Szantoi et al., 2013)	$E_{\text{NT}}=-\sum_{i}\sum_{j}P\left(i,j\right)\text{log}\left(P\left(i,j\right)\right)$	Indicates the randomness of the amount of information contained in the image
Angular Second Moment(Asm) (Szantoi et al., 2013)	$A_{\text{SM}}=\sum_{i}\sum_{j}P{\left(i,j\right)}^{2}$	Indicates the uniformity of the gray level distribution of the image and the thickness of the texture
Correlation(Cor) (Szantoi et al., 2013)	$C=\sum_{i=0}^{\text{quank}}\sum_{j=0}^{\text{quantk}}\frac{\left(i-M_{\text{ean}}\right)\times \left(j-M_{\text{ean}}\right)\times P\left(i,j\right)}{V_{\text{ar}}}$	Indicates the similarity of image gray levels in the row or column direction

#### 3.3.3 Boruta feature selection

Boruta is a feature selection algorithm designed to help identify the most important features in a dataset. It is based on the idea of random forests and is able to handle datasets with high-dimensional feature spaces (Subbiah and Chinnappan, 2021).The Boruta algorithm progressively distinguishes between important and unimportant features by means of continuous iteration until a pre-determined number of feature selections or some stopping criterion is reached. Eventually a result ranking the importance of the features will be given. This method is able to find the most distinguishable features without *a priori* information and is very effective when dealing with high dimensional datasets. This study is based on R4.3.0 to accomplish Boruta Feature Selection.

#### 3.3.4 Three-dimensional spectral characterization

The basic principle of the BCI method is to combine any three bands to construct three-dimensional spectral indices (Dou et al., 2023). Since the hyperspectral data cover hundreds of bands, there must be redundancy in the large amount of information, in order to streamline the information and effectively reduce the data dimensions, this study uses the BCI method to construct the optimal band feature combinations. The principle of this method is that the smaller the correlation between bands, the larger the standard deviation of the bands, the larger the information content of the band combination. The information content of the waveband combination is inversely proportional to the correlation coefficient between wavebands and directly proportional to the standard deviation of the wavebands themselves. By traversing the 272 bands of the 20th order differential and selecting the optimal wave combinations, the following six three-band indices are calculated Equations (2–7):


(2) $$\begin{array}{c} TBI_{1}\left(R_{i},R_{j},R_{k}\right)=\frac{R_{i}}{\left(R_{j}\times R_{k}\right)} \end{array}$$

(3) $$\begin{array}{c} TBI_{2}\left(R_{i},R_{j},R_{k}\right)=\frac{R_{i}}{\left(R_{j}+R_{k}\right)} \end{array}$$

(4) $$\begin{array}{c} TBI_{3}\left(R_{i},R_{j},R_{k}\right)=\frac{\left(R_{i}-R_{j}\right)}{\left(R_{j}+R_{k}\right)} \end{array}$$

(5) $$\begin{array}{c} TBI_{4}\left(R_{i},R_{j},R_{k}\right)=\frac{\left(R_{i}-R_{j}\right)}{\left(R_{j}-R_{k}\right)} \end{array}$$

(6) $$TBI_{5}\left(R_{i},R_{j},R_{k}\right)=\frac{\left(R_{j}+R_{k}\right)}{R_{i}}$$

(7) $$\begin{array}{c} TBI_{6}\left(R_{i},R_{j},R_{k}\right)=\frac{\left(R_{i}-R_{j}\right)}{\left[\left(R_{i}-R_{j}\right)-\left(R_{j}-R_{k}\right)\right]} \end{array}$$

Where *R_i_*, *R_j_*, *R_k_* are the reflectance values for all possible bands *i*, *j*, *k* in the wavelength range of 400−1000 nm with i≠j≠k . In this study, the construction of band indices was implemented in PyCharm 2022 software.

### 3.4 Predictive modeling and evaluation indicators

#### 3.4.1 Modeling strategy

**Figure 2:**
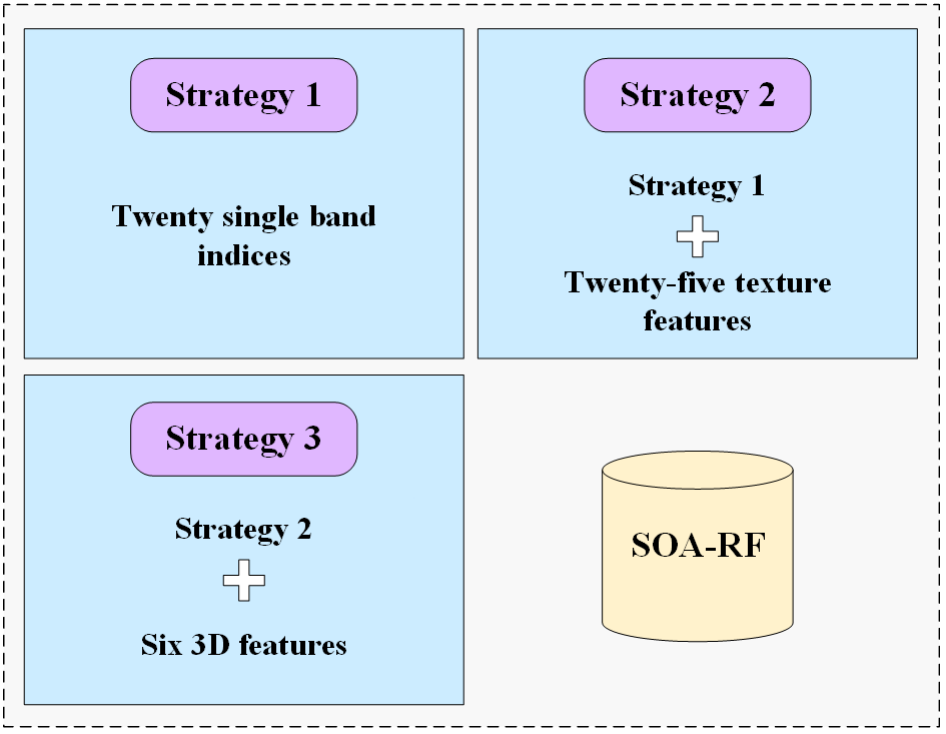
Modeling Strategies for Soil Salinity Estimation Models.

To assess the significance of various characteristic variables and their impact on the model’s sensitivity, this study devised three strategies for constructing the model (**Figure 2**). Strategy 1 involves constructing the model solely with the 20 single-band variable inputs screened by the CARS algorithm. In Strategy 2, 25 texture features augment Strategy 1, resulting in 45 feature variables for model construction. Strategy 3, building upon Strategy 2, incorporates six triple band indices, totaling 51 variables used in the model’s construction. Comparing these three strategies enables a discussion to investigate the contribution of distinct characteristic variables to the estimation model.

#### 3.4.2 Combined with seagull optimization algorithm for random forest models

SOA is a bionic intelligence algorithm that simulates the foraging behavior of seagulls. It optimizes the performance of the algorithm through the search and learning process in the foraging behavior (Dhiman et al., 2021). SOA is used to optimize the parameter selection and feature selection of the RF algorithm. Specifically, SOA improves prediction accuracy by tuning parameters in the RF algorithm, such as the number and depth of decision trees. Also, SOA reduces the impact of redundant features on the prediction results by selecting the best subset of features.

#### 3.4.3 Evaluation indicators

In this study, three evaluation metrics are used to assess the performance of our model, which include root mean square error (RMSE), mean square error (MSE), relative analytical error (RPD), and coefficient of determination (R^2^). Among these metrics, smaller values of RMSE and MSE, larger values of RPD along with R^2^, and R^2^ close to one, all represent high accuracy of the inverse model. Conversely, larger values of RMSE and smaller values of RPD and R^2^ imply lower accuracy of the model. These metrics will help us to comprehensively assess the performance of the constructed model and ensure the reliability of the research results.

## 4 Results

### 4.1 Statistical analysis of soil samples

**Figure 3:**
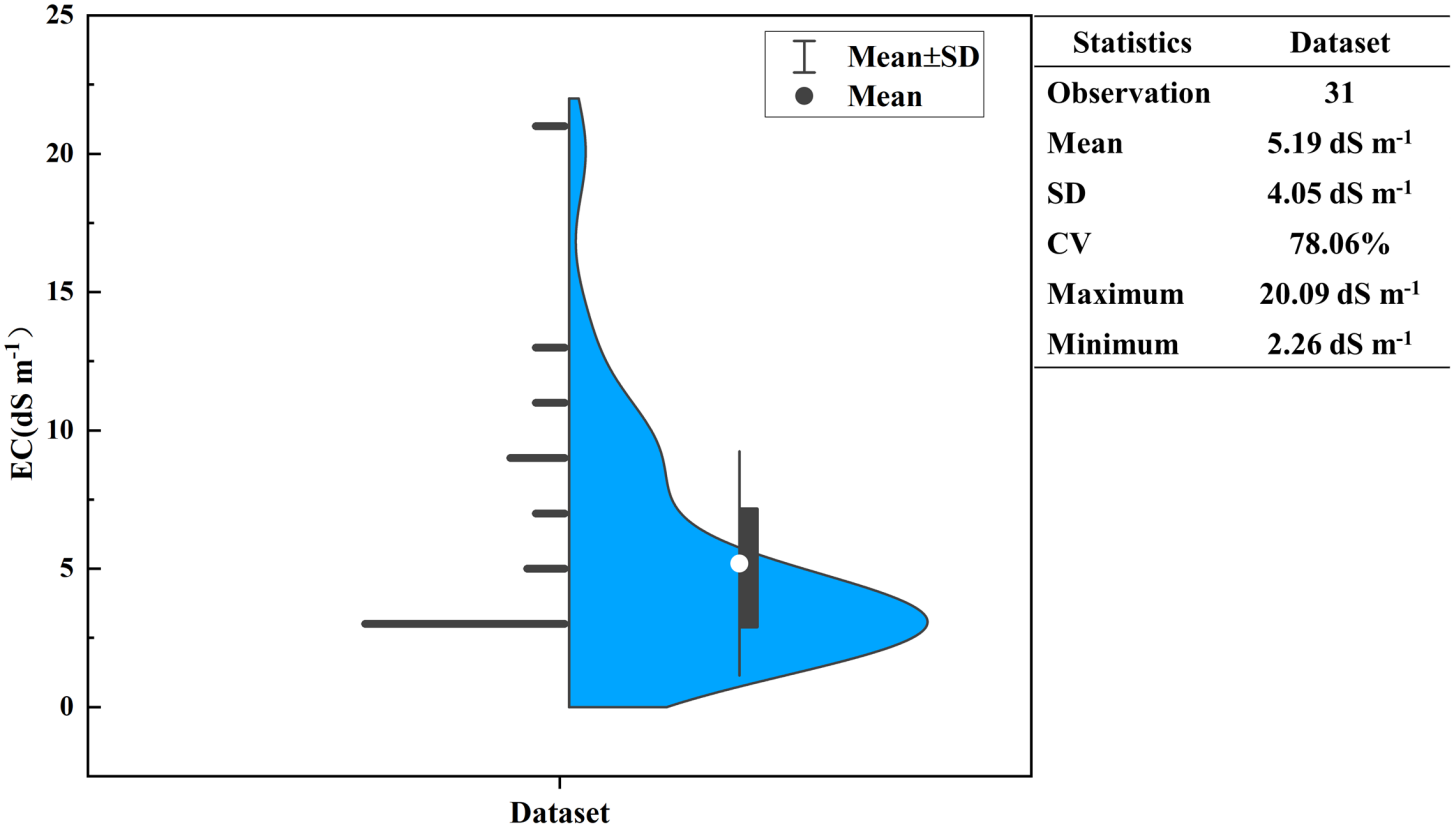
Soil salinity samples and their descriptive statistics (blue area shows the kernel density distribution of the samples).

Descriptive statistics of 33 soil samples, as shown in **Figure 3**, showed that the EC_1:5_ of the samples ranged from 2.26 dS m^-1^ to 20.09 dS m^-1^, with a mean of 5.19 dS m^-1^, a standard deviation of 4.05 dS m^-1^, and a coefficient of variation (CV) of 78.06% (10%<CV<90%, which is a moderate variation), which suggests that the data have a sufficiently discrete degree for model construction and prediction.

### 4.2 Spectral response characteristics

**Figure 4:**
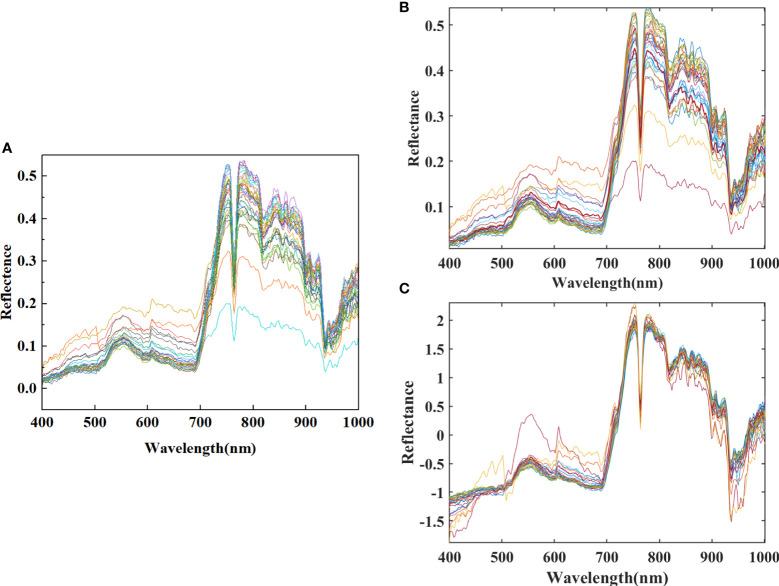
**(A)** The original spectrum, **(B)** the spectrum after Savitzky-Golay filtering and **(C)** the spectrum after Standard Normal Variate (SNV) processing.

Comparing the original spectrum (**Figure 4A**), the SG-filtered spectrum (**Figure 4B**) and the SNV-treated spectrum (**Figure 4C**), the influential noise is further eliminated after SG smoothing, and the spectral reflectance range is expanded to -2 to 2.5, which well highlights the spectral characteristics of cotton. Soil salinity mainly images cotton’s absorption of water and nitrogen, which in turn affects chlorophyll synthesis, and the degree of salinity leads to differences in the degree of reflection and absorption of electromagnetic waves in the bands at 555 nm and 680-690 nm affected by chlorophyll, and also affects the reflectance intensity of the red-edge bands, as well as the degree to which the electromagnetic waves at 763 nm, 821 nm, and 935 nm are absorbed by water.

### 4.3 FOD treatment results

**Figure 5:**
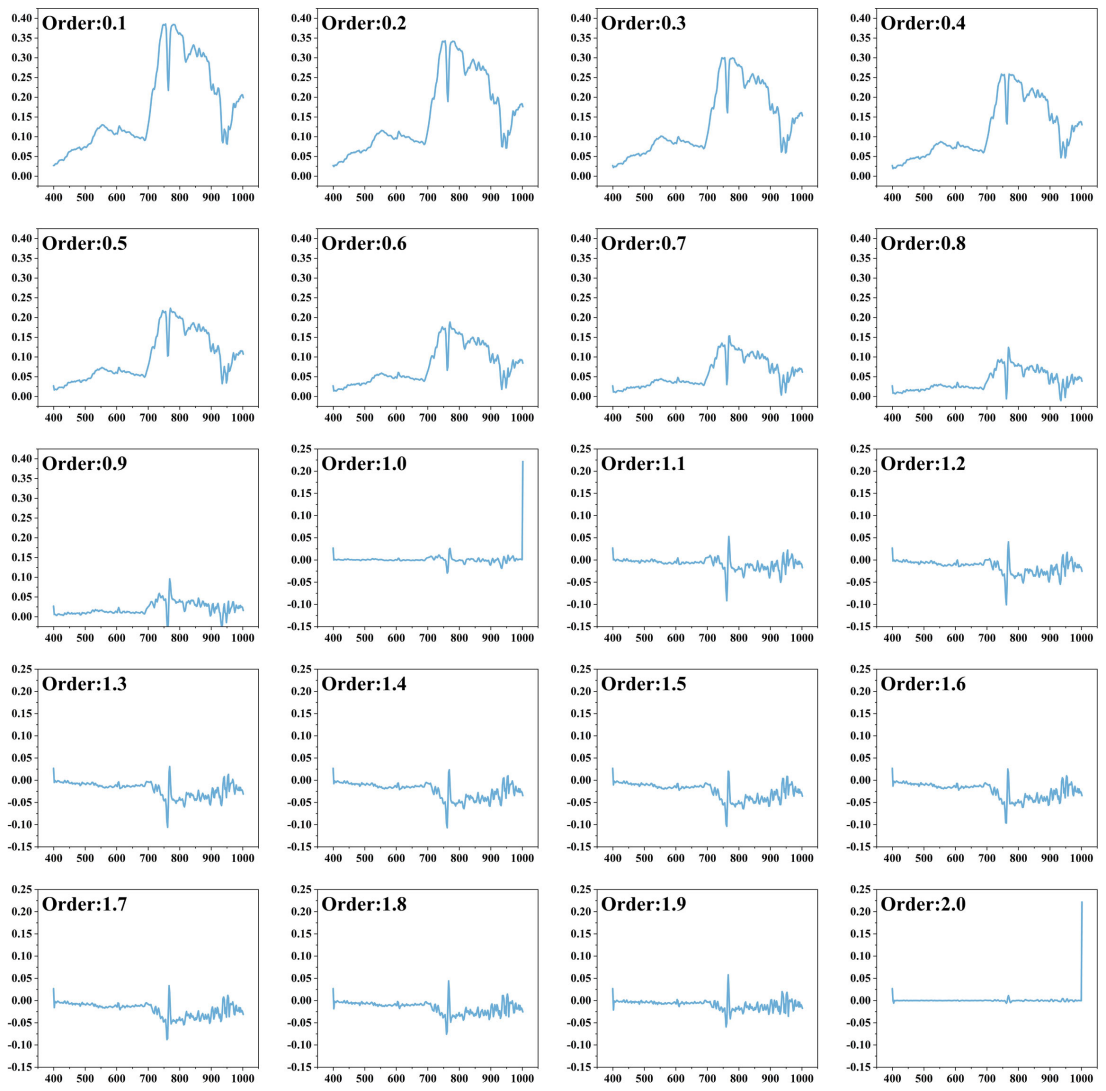
0.1~2.0 Order Differential Spectral Curves (X-axis is wavelength, Y-axis is reflectance).

The soil reflectance spectra were processed by FOD with the order of 0-2 and the step size of 0.1, and the processed results are shown in **Figure 5**. With the increase of FOD order, the values in the whole spectral reflectance range gradually decrease and converge to 0. The small differences between spectra are highlighted to avoid the loss of important information and enhance the effect of data preprocessing. In the 0.1-1 order spectra, positive peaks appear at 506 nm, 606 nm and 770 nm, and negative peaks appear at 761 nm and 932 nm; in the 1-2.0 order spectra, positive peaks appear at 501 nm, 766 nm and 939 nm, and negative peaks appear at 759 nm and 930 nm.

### 4.4 Construction of the characteristic index

#### 4.4.1 Single-band feature construction

**Figure 6:**
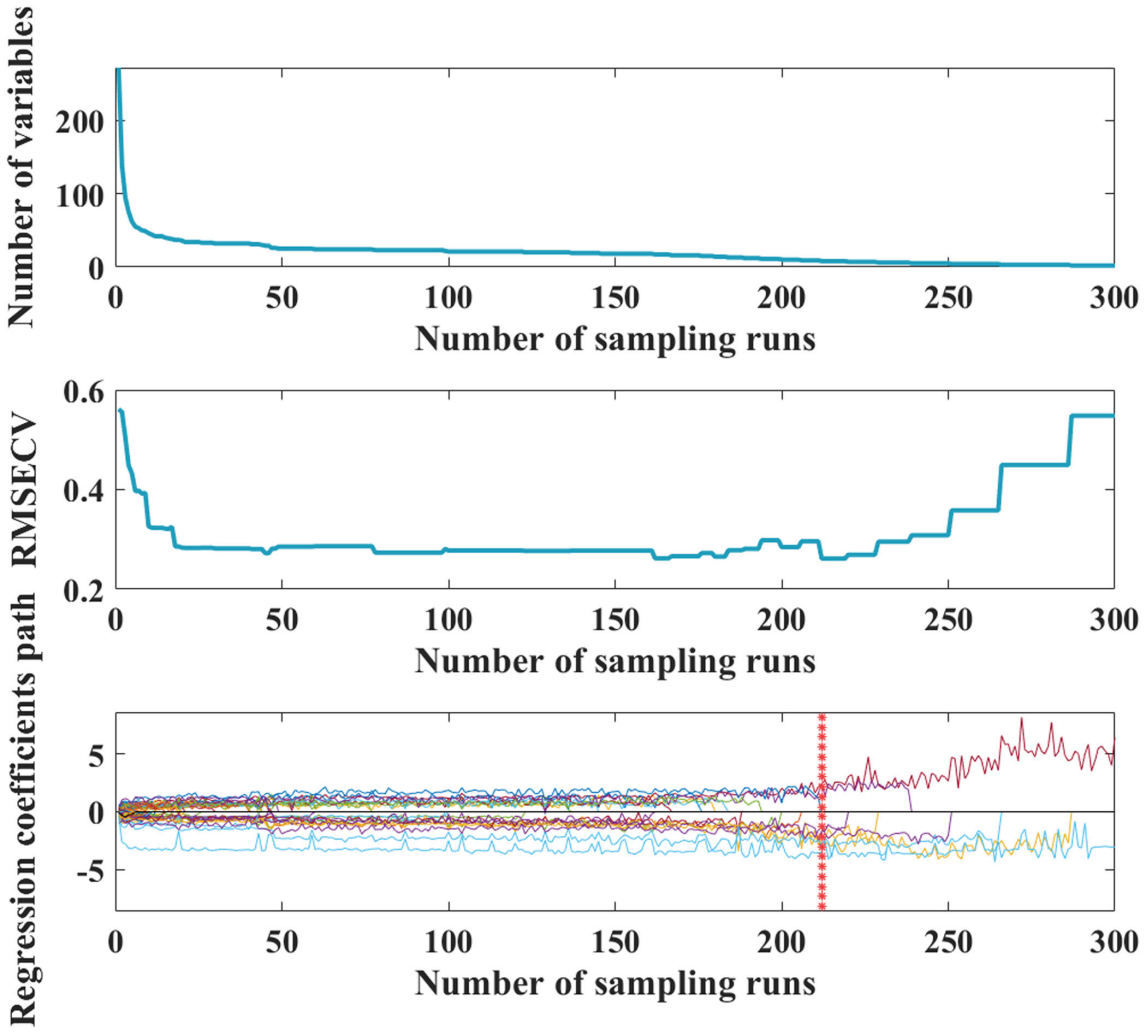
competitive adaptive weighted sampling (CARS) process.

**Figure 7:**
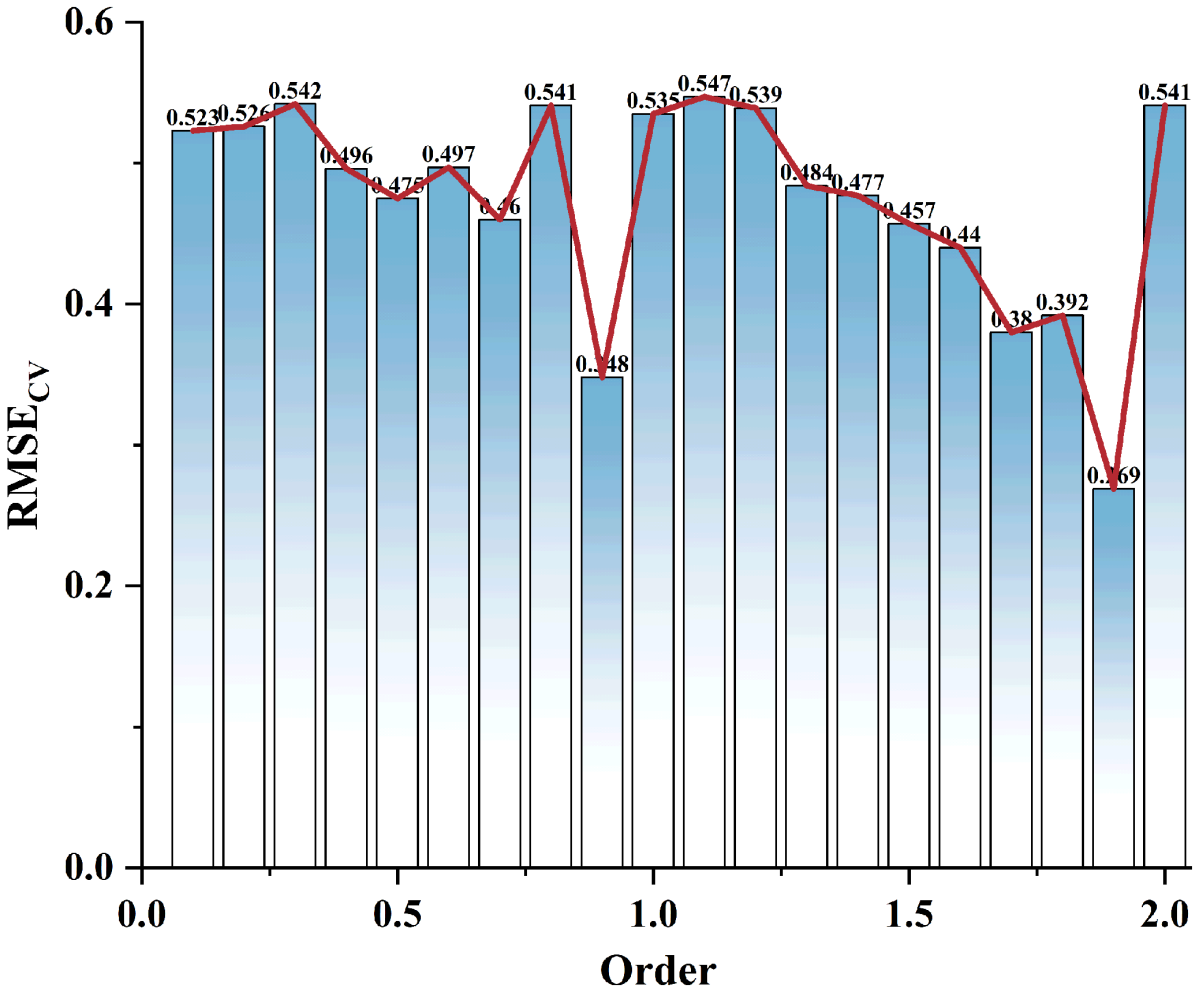
RMSE_CV_ values for 0 to 2.0 order differentiation.

Firstly, the spectral information of 0.1-2.0 order differentiation is used for the selection of single band eigenvariable by CARS, the number of iterations is set to 300, and the data is centered and the step size is set to 10. The process of CARS processing is shown in **Figure 6**, with the increase of the number of iterations, the number of selected bands decreases, and the RMSE_CV_ decreases gradually, and when the number of iterations is 216, the RMSE_CV_ reaches the minimum value. The result can be seen that the best FOD order for CARS screening is 1.9, and the minimum RMSE_CV_ is 0.269 (**Figure 7**), at this time, the best band with the maximum amount of information is determined, that is the requested feature band.

**Figure 8:**
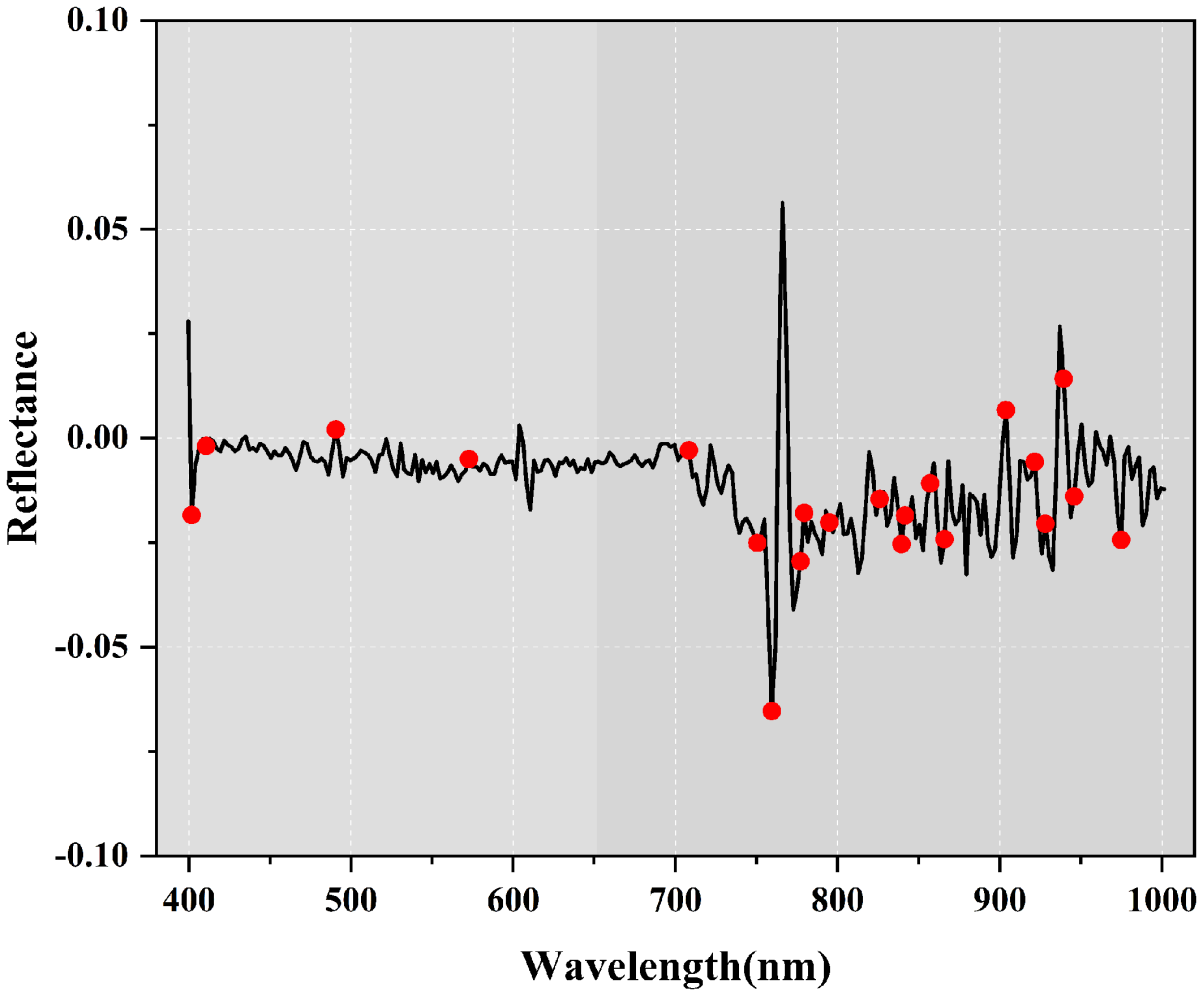
Optimal bands obtained from Competitive adaptive reweighted sampling (CARS).

A total of 20 best characteristic bands were screened out, which only accounted for 7.35% of the full spectral bands, but did contain the maximum amount of information that could express the relationship between soil salinity and spectra. 20 characteristic bands were labeled in **Figure 8**, of which eight bands were located in the visible spectra (400~760 nm), and 12 bands were located in the near-infrared spectra (760~1000 nm).

#### 4.4.2 Construction of texture features

**Figure 9:**
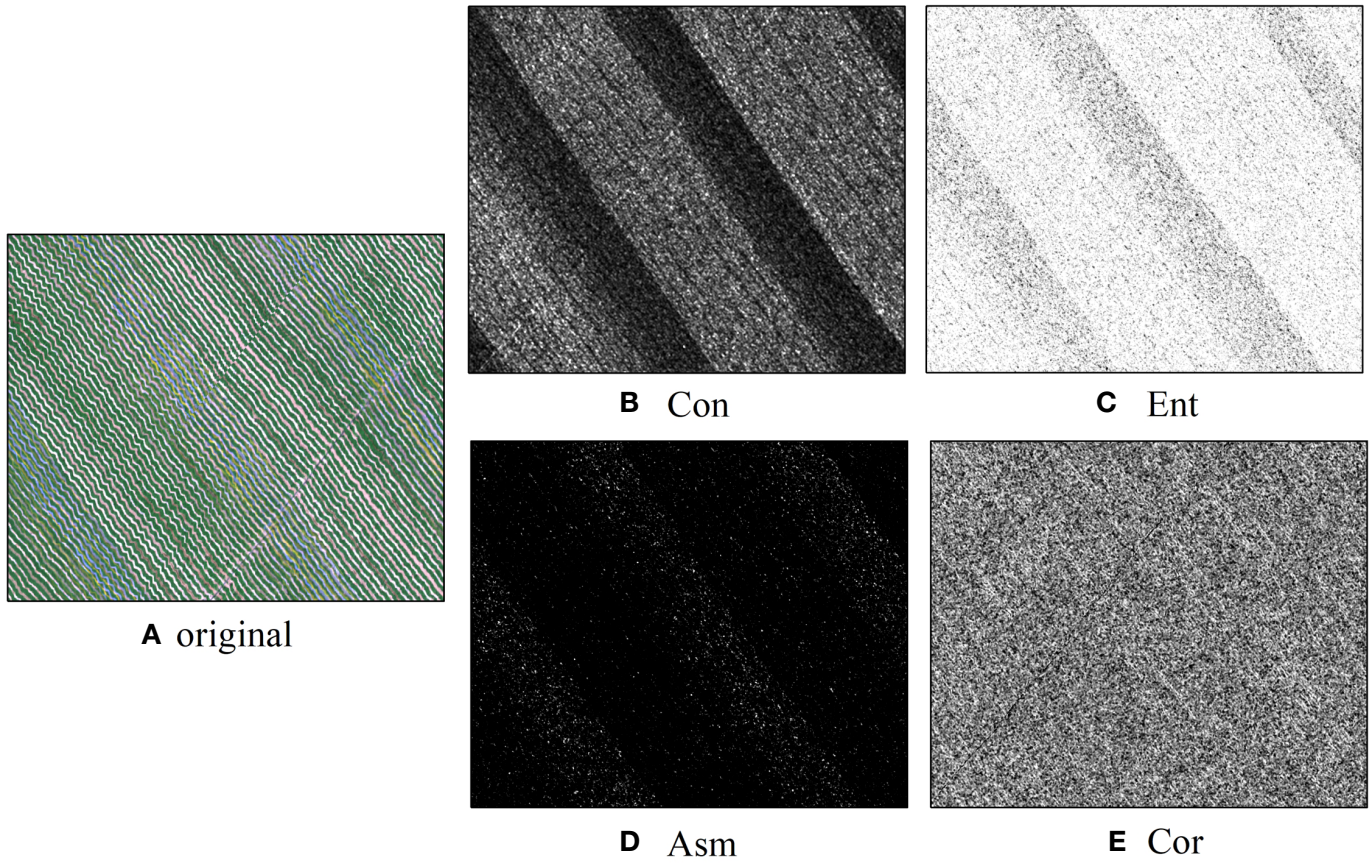
Texture characteristics [**(A)** Original; **(B)** Contrast (Con); **(C)** Entroy (Ent); **(D)** Angular Second Moment (Asm); **(E)** Correlation (Cor)].

The four features of contrast, entropy, angular second order distance and correlation calculated using the gray scale covariance matrix were used to characterize the texture (**Figure 9**). According to the results of single-band screening conducted by CARS, the best order of FOD is 1.9, which shows that 1.9 order differential spectra can better characterize the correlation information features with EC_1:5_, so in this study, we calculate the grayscale co-generation matrix for the 20 bands screened by CARS from the 1.9 order differential spectra, and the window size of the grayscale co-generation matrix is selected to be 5×5, and the direction is selected to be x=2 and y=2, which yields 80 texture features.

**Figure 10:**
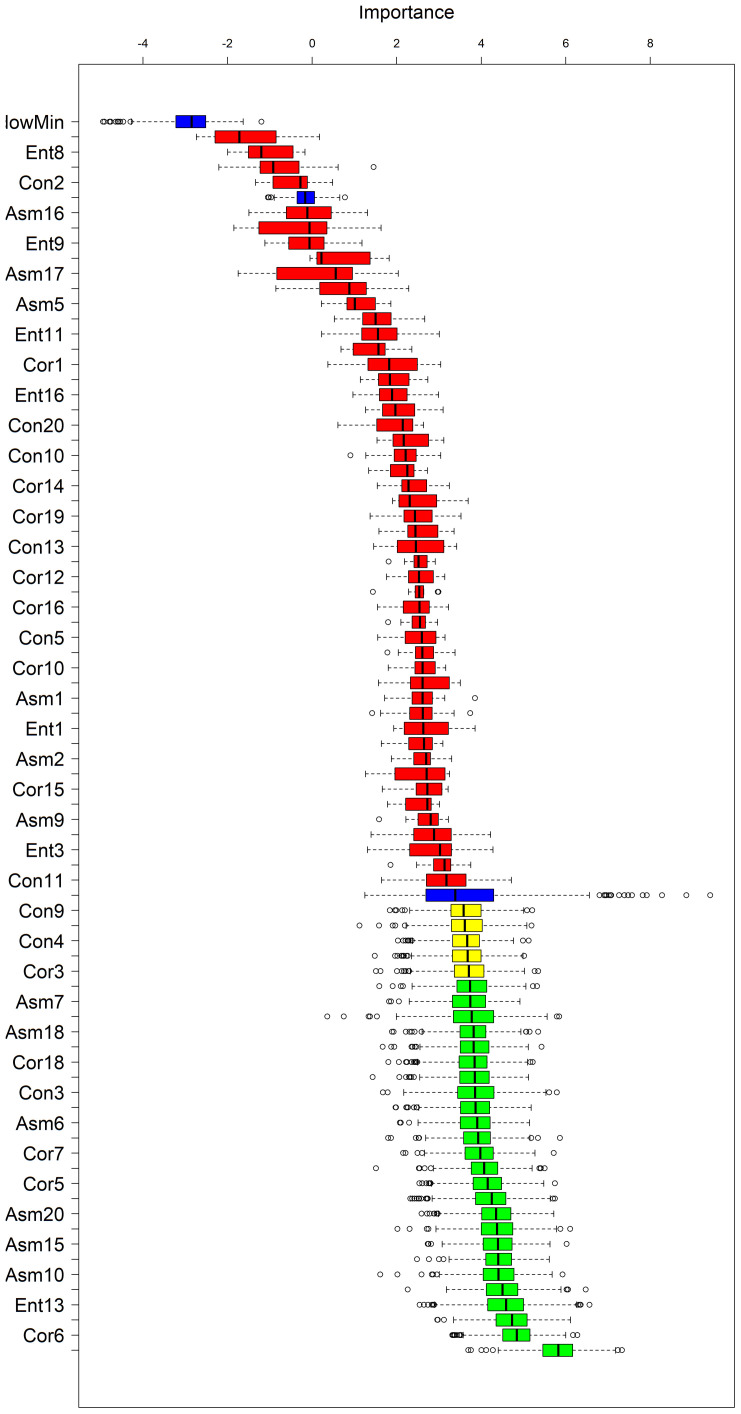
Boruta algorithm screening results (The blue variables are shaded features, the green variables are essential, the yellow ones are undetermined, and the red ones are irrelevant).

In order to avoid model overfitting with too many feature variables, the Boruta algorithm was used to filter out unimportant feature values. The results show that 25 of the 80 texture features are very important, including 6 contrast variables, 3 entropy variables, 11 angular second-order moment variables and 5 correlation variables; the remaining are 5 generally important and 50 unimportant variables. In this study, 25 very important texture features were selected to participate in the modeling strategy (**Figure 10**).

#### 4.4.3 Construction of 3D features

CARS selects characteristic bands from spectra only from a one-dimensional perspective to seek the relationship between spectral information and soil salinity, and in order to consider the interaction between bands and soil salinity, the BCI method is utilized to construct a three-dimensional band index to explore the relationship between spectral information and soil salinity in a more comprehensive way.

**Figure 11:**
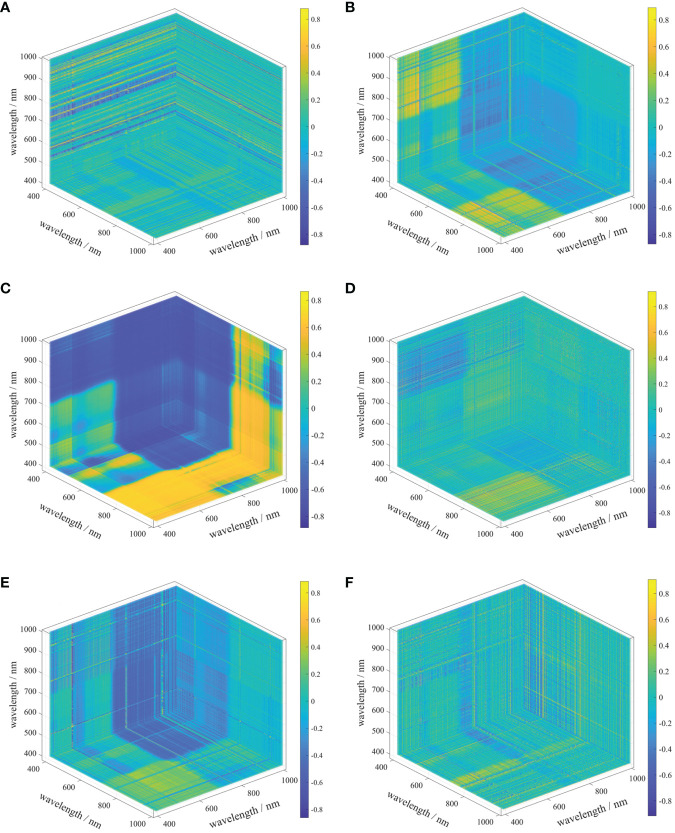
Three-dimensional band index [**(A–F)** are TBI1, TBI2, TBI3, TBI4, TBI5, TBI6 respectively].

The 3D spectral indices in different order spectral forms were calculated and the correlation coefficients between the indices and soil EC_1:5_ were calculated and the results are shown in **Table 3** and finally the most sensitive way of combining the bands with EC_1:5_ was selected for the different orders TBI1, TBI2, TBI3, TBI4, TBI5 and TBI6. The 3D cubic plot of the correlation coefficients is shown in **Figure 11**.

**Table 3:** Optimal Differential Order and Band Combinations for Constructing Three-Dimensional Features(R is the correlation coefficient).

3D index	Optimum order	wave portfolio(nm)	R
TBI1	1.0	423.957,506.137,777.108	0.868
TBI2	0.9	421.736,865.952,996.995	0.891
TBI3	0.6	401.746,945.910,421.736	0.865
TBI4	1.8	421.736,526.126,925.921	0.912
TBI5	0.9	415.072,521.684,934.805	0.885
TBI6	1.0	423.957,954.795,923.700	0.891

### 4.5 Modeling and evaluation

**Figure 12:**
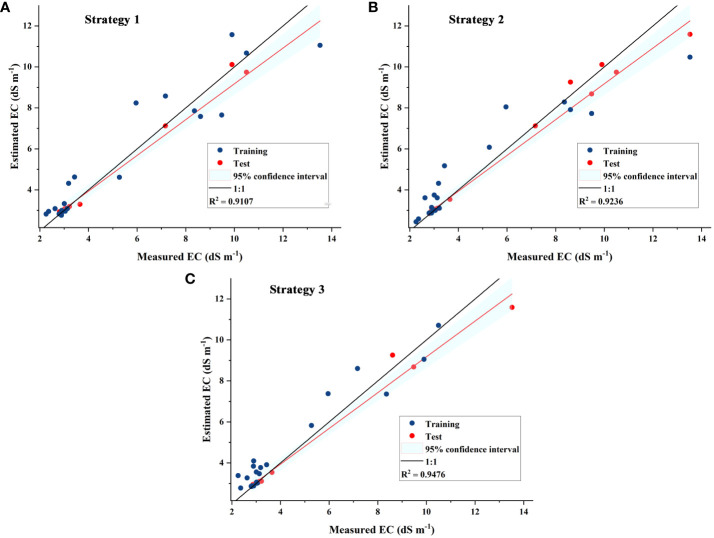
Scatter plots of measured and estimated EC in **(A)** Strategy 1, **(B)** Strategy 2 and **(C)** Strategy 3.

The soil samples were divided into training set and test set input models according to 7:3, and the SOA-RF performance results of the three modeling strategies are shown in **Table 4** and **Figure 12**, which shows that the R^2^ and RPD of the three modeling strategies gradually increase, the RMSE and MSE gradually decrease, and there is a significant improvement in the modeling accuracy of modeling strategy III. At the same time, the MSE of SOA-RF and traditional RF models are compared, and the MSE of the three modeling strategies of SOA-RF are much smaller than those of RF models, which shows that the introduction of SOA algorithms improves the model performance greatly.

**Table 4:** SOA-RF model performance (MSE left side indicates SOA-RF, right side indicates RF).

Modelling strategy	R^2^	RMSE (dS m^-1^)	RPD	MSE (dS m^-1^) (SOA-RF/RF)
Strategy 1	0.9107	1.4707	1.8736	2.1630/15.3478
Strategy 2	0.9236	1.3917	2.6854	1.9367/15.2769
Strategy 3	0.9476	1.2554	3.5223	1.5761/12.4969

**Figure 13:**
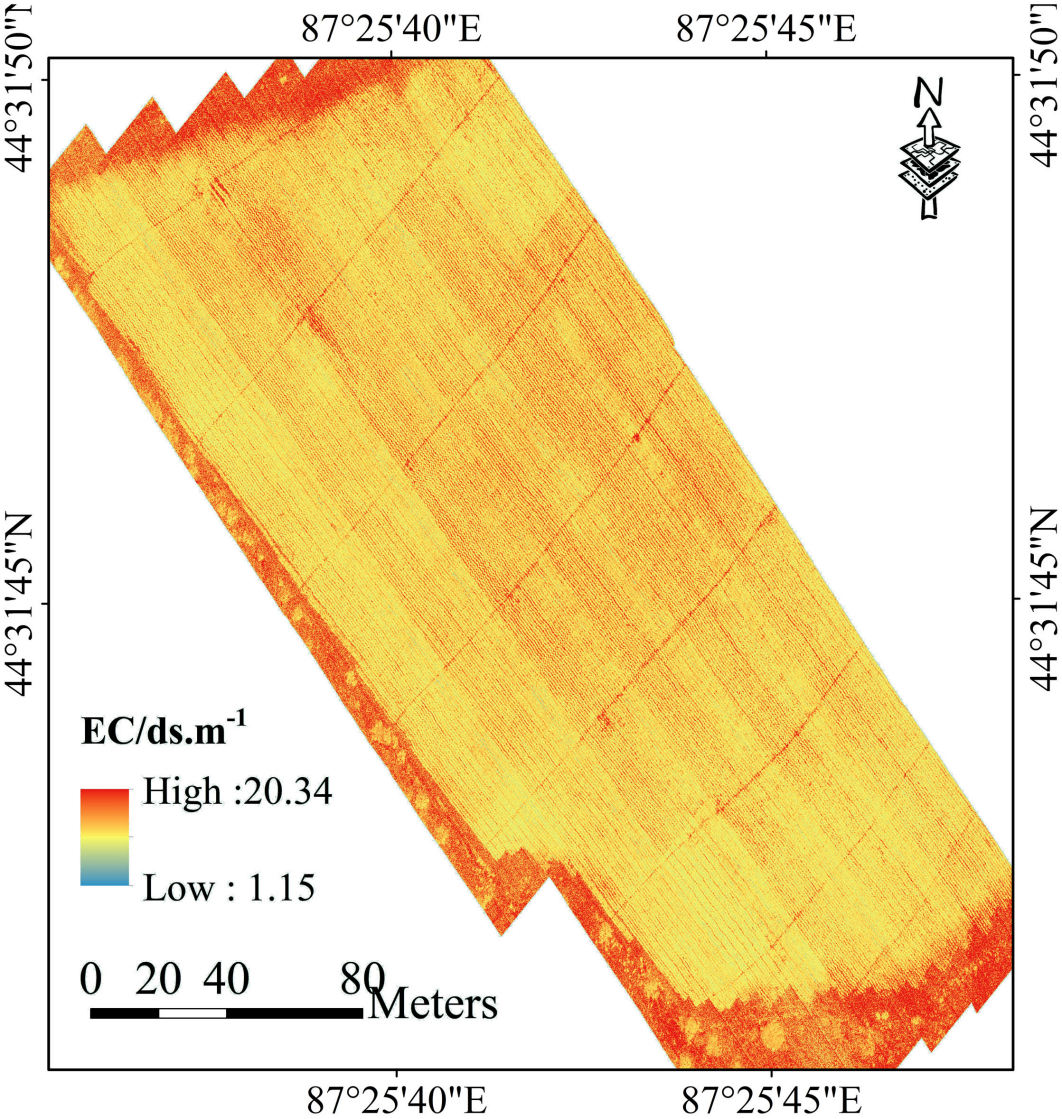
Soil Salinity (EC_1:5_) Mapping Based on Optimal Strategy and SOA-RF Modeling.

The spatial distribution of top soil salinity in cotton fields was mapped using the SOA-RF model (**Figure 13**). From the descriptive statistical analysis conducted by EC_1:5_, the measured EC_1:5_ of the soil ranged from 2.26 dS m^-1^ to 20.09 dS m^-1^, and the optimal model predicted soil salinity ranging from 1.15 dS m^-1^ to 20.34 dS m^-1^, and the spatial distribution of soil salinity was in accordance with the results of the field survey. Sub-membrane drip irrigation technology provides a breakthrough for the sustainable development of agro-ecology in this region. Sub-membrane drip irrigation technology can achieve the purpose of water-saving irrigation, but the small amount of irrigation water is difficult to adequately wash the salts in the soil, and the strong evapotranspiration in this region has been continuous, which makes the salts in the soil will always be migrated to the surface of the soil, so that the salts in the surface layer of the soil accumulates year by year, especially when the soil is not covered with a membrane it is very obvious to see that there are soil salts higher than the plant salinity, so that the salts in the surface layer of the soil are higher than the plant salts. The soil salinity is higher than that of the vegetated areas, especially in the case of non-mulched areas.

## 5 Discussion

### 5.1 Importance of fractional order differential treatment

Preprocessing of hyperspectral data is the most important step before building quantitative prediction models (Minu et al., 2016). In this study, the hyperspectral data were first subjected to S-G smoothing and SNV processing, which clearly represented the spectral characteristics of cotton affected by salinity with abnormal changes at the level of spectral reflectance, and removed and amplified the spectral characteristics by removing the spectral signal variance.

**Figure 14:**
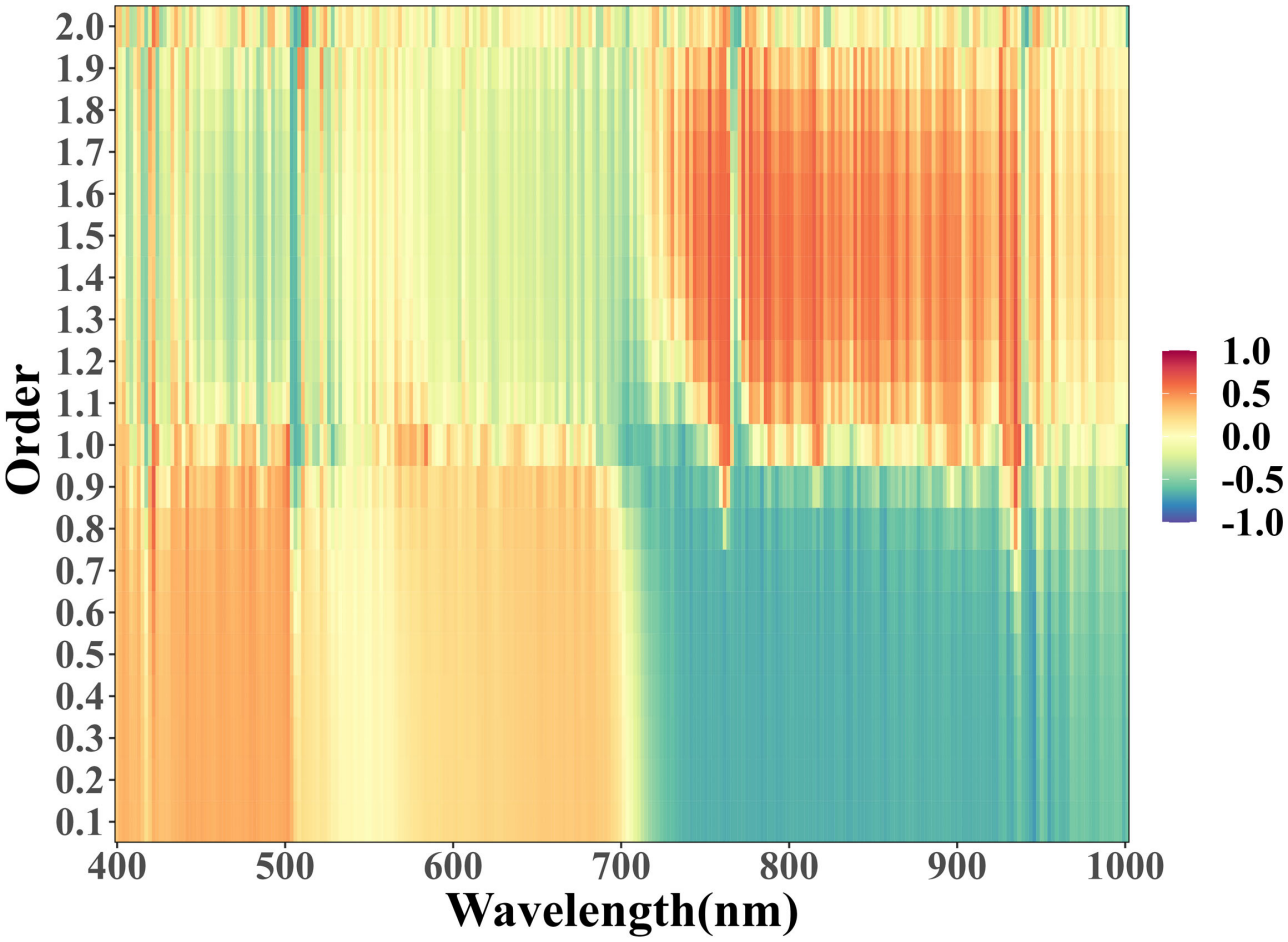
Thermogram of correlation between 0 to 2.0 order spectra and soil EC_1:5_.

However, hyperspectral data are complex and contain a large amount of intricate information and noise between consecutive and short spaced bands, which requires further fractional order differentiation processing of hyperspectral data (Sun et al., 2021). The results show that FOD is more helpful to reduce the sharp peak aberrations than integer-order differencing, which maintains the structure of the original spectral curves and preserves the original information, but also makes the baseline vary within a moderate curvature range, which makes more useful information of the spectral data to be highlighted (Hong et al., 2020). In this study, we differentiated the hyperspectral data by 20 orders at 0.1 intervals and found that the FOD spectra could reveal hidden information related to the vegetation spectral information. A correlation heat map of the 0 to 2.0 order spectra with soil EC_1:5_ was plotted (**Figure 14**), with wavelength on the horizontal axis, order on the vertical axis, and the color representing the magnitude of the correlation coefficient. The correlation heat map shows a nearly symmetrical pattern, when the FOD order is between 0.1 and 1, the reflectance in the wavelength range of 400-700 nm is basically positively correlated with soil EC_1:5_, and the reflectance in the wavelength range of 700-1000 nm is basically negatively correlated with soil EC_1:5_; when the FOD order is between 1.0 and 2.0, the soil reflectance in the range of 400-700 nm shows a weak correlation with soil EC_1:5_. When the FOD order is between 1.0 and 2.0, the reflectance of soil in the range of 400-700nm shows a weak positive or negative correlation; the reflectance of soil in the range of 700-1000nm basically shows a positive correlation. With the increase of FOD order, the correlation of reflectance at the blue light wavelength is gradually weakened, the negative correlation of reflectance at the red light and near-infrared wavelengths is changed to a positive correlation with the division of 1.0 order, but the overall correlation is strong, and the weak correlation of reflectance at the green light wavelengths tends to be stabilized. The core purpose of FOD processing is to bring out the useful information from the complex and noisy hyperspectral data, and the subsequent processing of the FOD processed data will help to improve the accuracy of the model, but for different research areas and different data, the FOD processing may present different effects and even results, and the method needs to be further investigated and improved in a variety of different research areas and using different data sets.

### 5.2 Impact of different spectral features on prediction models

Extracting appropriate feature variables from hyperspectral data containing a large amount of information is a key step in constructing a stable model (Lao et al., 2021), and the three modeling strategies can be compared to derive the degree of sensitivity of the three feature indicators.

Modeling strategy I used only the single-band feature variables screened by CARS to participate in the modeling, and the results showed that the 1.9 order was the optimal differential order which was the same as the optimal differential order for region I in the study of salinity content estimation in different saline zones by (Fu C. et al., 2021). The characteristic bands were scattered in the visible (408-777 nm), but mainly concentrated in the near-infrared (NIR) shortwave (800-1000 nm), which indicated that the NIR bands performed better in the characterization of soil salinity using vegetation spectra, which was also confirmed in the results of (Tian et al., 2021) and (Nguyen et al., 2020). However, since the single-band variables can only reflect the spectral properties from a one-dimensional perspective, the R^2^ of modeling strategy I is only 0.9107, with the lowest coefficient of determination among the three modeling strategies, and the RPD is 1.8736, which is also the weakest in terms of stability.

Modeling strategy II adds 25 texture features to the single-band variables, and the results show that modeling strategy II improves R^2^ by 0.0129 and RPD by 0.8118.The overall improvement of R^2^ is not significant, which may be due to the fact that texture features are also statistics of single-band information (Zhang et al., 2018), and although texture features increase the spatial distribution describing spatial dimensionality information, it lacks the characterization of interconnected information between bands (Fu Y. et al., 2021). The RPD enhancement is more obvious compared to R^2^, and the stability of the model is significantly improved by adding texture features. Overall, the combination of single-band indices with image texture parameters can increase the dimensionality of the information, which can more accurately characterize yield variations, which is consistent with the findings of (Wang et al., 2021) in the performance of spectral indices alone and in combination with spectral indices based on texture measurements of UAV-based hyperspectral images in seed yield estimation. However, the inclusion of texture features provided less overall improvement to the prediction model.

**Figure 15:**
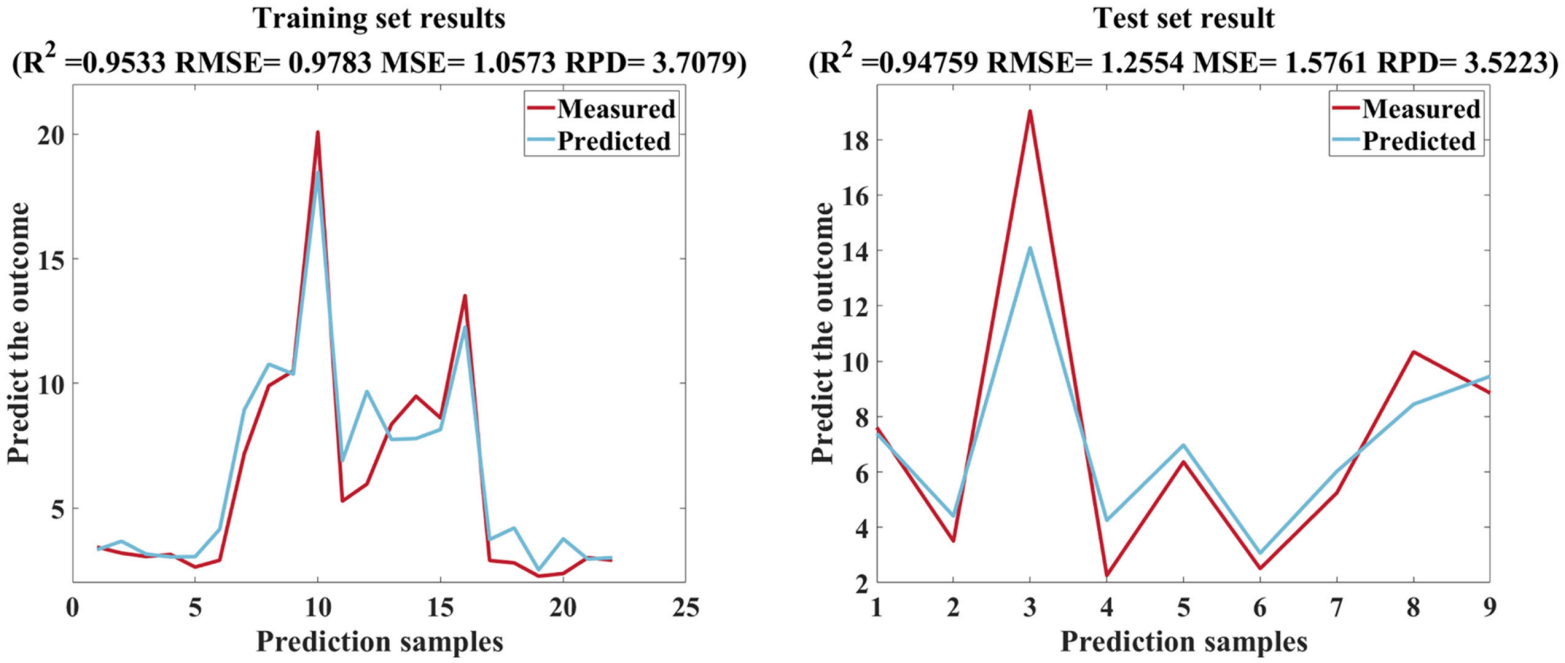
SOA-RF training set and test set results.

Modeling strategy three adds six three-band indices to strategy two, and the results show an improvement of 0.0369 in R^2^ and 1.6478 in RPD compared to strategy one, which is a very significant overall improvement (**Figure 15**). The three-band index improves the model performance more than the single-band index, which is consistent with the conclusion that the three-band index constructed by OBCA in the study of (Zhu et al., 2022) improves the relationship between spectra and soil salinity, which is of high importance in modeling, and this study is the first time that texture features are added into the modeling strategy to compare with the three-band index, and it is seen that the three-band index is also more sensitive than the texture features are more sensitive. The correlations between the six tri-band indices and soil EC_1:5_ were similar in magnitude, with TBI4 showing the best performance with an R of 0.912, and it can also be found that all six tri-band index band combinations contain bands within 777~1000 nm, which shows that for the tri-band indices, the near-infrared band is still the most sensitive band to soil EC_1:5_, which is also consistent with the findings of (Wang S. et al., 2019) study in which the selected characteristic bands using the SPA method showed that different spectral transformation forms characterize soil salinity differences, mainly in the NIR band in agreement.

The screening and construction method of feature indices used in this study can extract effective spectral variables from highly redundant hyperspectral data while ignoring most irrelevant variables, thus substantially improving the accuracy of the model. However, there may be different results for different crop types and regions, and further research on the generalizability of the method is needed.

### 5.3 Advantages of the SOA-RF model

**Figure 16:**
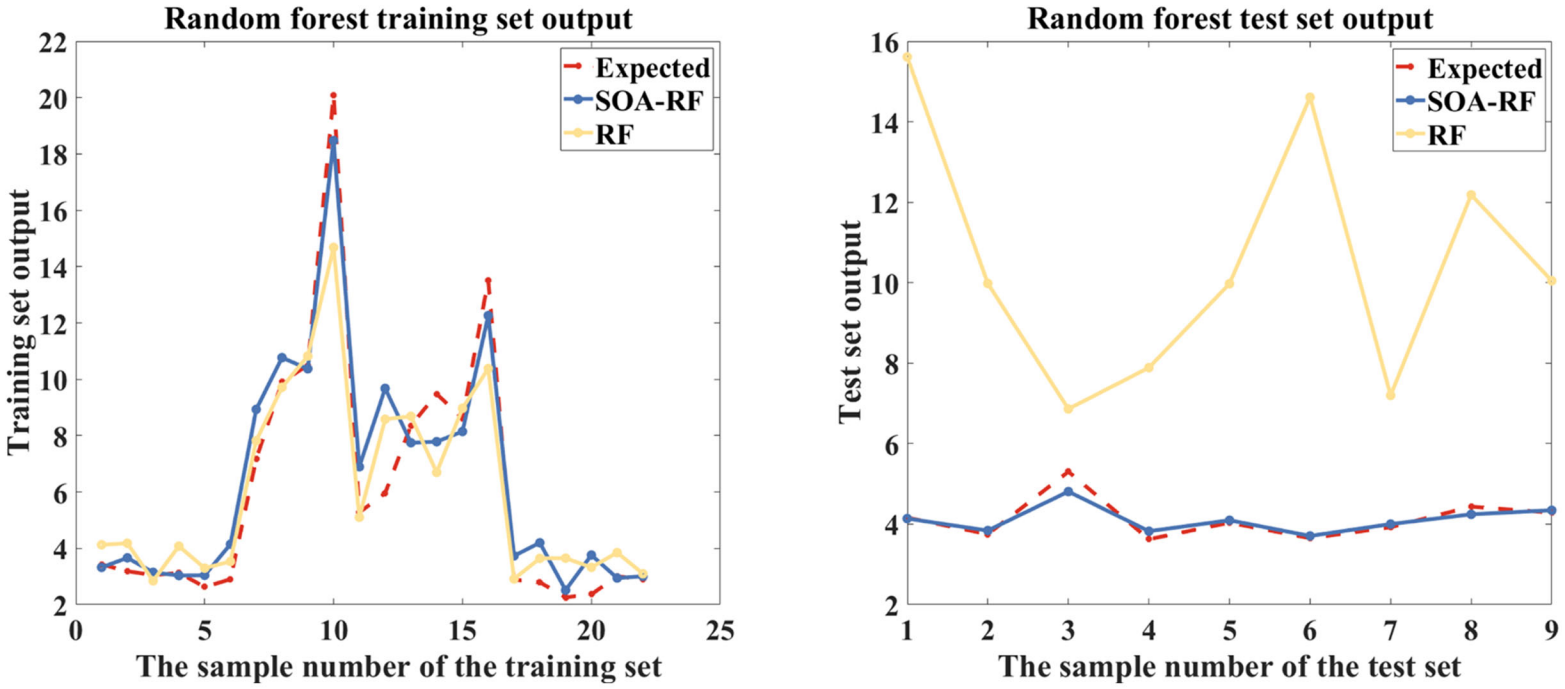
Comparison of SOA-RF and RF output results.

There are many studies that use machine learning methods for soil salinity prediction (Zhu et al., 2022), utilized RF model R^2^ to reach 0.815 in a study of soil salinity inversion of Abbey Lake Oasis based on UAV hyperspectral imagery; (Jia et al., 2022) used an RF model with an R^2^ of 0.93 in a study of salinity inversion for different cultivated soil types based on hyperspectral data and machine learning; (Hu et al., 2019) reached an R^2^ of 0.94 in quantitative soil salinity estimation using RF model predictions based on UAV hyperspectral data; (Cui et al., 2023) used unmanned aerial multispectral remote sensing and three machine learning algorithms to invert the soil salinity of farmland at different depths under crop cover, and the optimal prediction model had an R^2^ of 0.775. After comparison, the accuracy of the SOA-RF model used in this study exceeds that of the previous models used (Zhao et al., 2022). used SOA algorithm to optimize the RF model in the study of vulnerability of agricultural soil and water resources system, and the R^2^ reached 0.9999; (Li et al., 2023) in the prediction of concrete compressive strength showed that the SOA-RF model outperformed ANN, ELM and empirical models; (Zhou et al., 2023) used the SOA-RF model in a study of maximum surface settlement prediction with an R^2^ of 0.9372. In this study, the SOA-RF model was innovatively used for the inversion of soil EC_1:5_, and the parameters of the RF model were optimized using the SOA algorithm, and there was a great improvement in the accuracy. A comparison of the outputs of the traditional RF and the SOA-RF model is shown in **Figure 16**, and although the traditional RF showed better results in the training set, the performance in the test set was very poor, and the suppression of the overfitting problem can be seen in the SOA-RF.

### 5.4 Research characteristics and limitations

To cope with the complex scenarios of vegetation and film cover during the cotton growing season, a method of quantitatively extracting soil salinity from cotton fields covered with film has been developed by combining spectral processing, band selection, spectral indices, texture features and machine learning algorithms, with the advantages of speed, simplicity, and non-destructive nature, with the main theme of “Salinity Information Extraction in Cotton Fields”, using hyperspectral remote sensing by unmanned aerial vehicle (UAV) as a means to enhance soil salinity monitoring in cotton fields, and providing a useful reference to optimize the irrigation technology in cotton fields.

Remote sensing by drones offers the possibility of obtaining crop growth information on a large scale and continuously. However, the area of remote sensing by drones is still limited, and future work needs to be carried out in conjunction with satellite remote sensing. And in the subsequent research, based on the UAV hyperspectral data, applying the developed optimal prediction model to map the soil salinity distribution in different fertility periods of cotton, we can try to explore the distribution law of soil salinity in different fertility periods of cotton, grasp the health status of cotton in different periods, put forward countermeasures to deal with excessive accumulation of soil salinity, and safeguard the cotton yield from the impact of soil salinity.

## 6 Conclusions

In this study, single-band features, texture features, and three-band features are constructed based on S-G, SNV, and FOD-processed hyperspectral data through a series of feature construction methods, and three modeling strategies are established using feature variables. The main results are as follows:

(1) The SNV treatment amplified the spectral properties and the correlation between soil EC_1:5_ and spectra varied with order.
(2) 1.9 order differentiation is the best differentiation order for constructing the single-band index; adding texture features can improve the estimation accuracy of the model, but the improvement of the accuracy is not obvious; after adding the three-band index, the estimation accuracy of the model is obviously improved, and the R^2^ reaches 0.9476.
(3) SOA-RF provides a dramatic increase in model accuracy and stability compared to traditional models.

The optimal soil salinity prediction model proposed in this study can accurately, non-destructively and quickly determine whether there is excessive salt accumulation in drip irrigation under the membrane, which is of guiding significance for the growth condition of cotton, the improvement of cotton yield and the sustainable development of the agricultural economy in Xinjiang, and also provides a reference for the regional prevention and control of soil salinization.

## Data availability statement

The original contributions presented in the study are included in the article/supplementary material. Further inquiries can be directed to the corresponding author.

## Author contributions

ZW: Writing – original draft, Writing – review & editing, Conceptualization, Methodology, Software, Visualization. JD: Writing – review & editing, Funding acquisition, Supervision. JT: Methodology, Software, Writing – review & editing. JL: Methodology, Software, Visualization, Writing – review & editing. TZ: Methodology, Writing – review & editing. WC: Formal Analysis, Writing – review & editing. SM: Software, Writing – review & editing.
